# An Approach to Describe *Salmonella* Serotypes of Concern for Outbreaks: Using Burden and Trajectory of Outbreak-related Illnesses Associated with Meat and Poultry

**DOI:** 10.1016/j.jfp.2024.100331

**Published:** 2024-07-18

**Authors:** Katherine E. Marshall, Zhaohui Cui, Brigette L. Gleason, Cassie Hartley, Matthew E. Wise, Beau B. Bruce, Patricia M. Griffin

**Affiliations:** Centers for Disease Control and Prevention, 1600 Clifton Rd NE, Mailstop H24-10, Atlanta, GA 30333, USA

**Keywords:** Foodborne disease outbreaks, Foodborne disease prevention, *Salmonella*

## Abstract

Over 40% of all U.S. *Salmonella* illnesses are attributed to consumption of contaminated meat and poultry products each year. Determining which serotypes cause the most outbreak illnesses associated with specific meat and poultry types can inform prevention measures. We developed an approach to categorize serotypes using outbreak illness burden (high, moderate, low) and trajectory (increased, stable, decreased). We used data from 192 foodborne *Salmonella* outbreaks resulting in 7,077 illnesses, 1,330 hospitalizations, and 9 deaths associated with chicken, turkey, beef, or pork during 2012–2021. We linked each meat and poultry type to 1–3 serotypes that we categorized as high outbreak illness burden and increased trajectory during 2021. Calculation and public display of outbreak illness burden and trajectory annually could facilitate the prioritization of serotypes for prevention by federal and state health and regulatory agencies and by the meat and poultry industry.

## Background

*Salmonella* is estimated to be the leading bacterial cause of U.S. foodborne illnesses, hospitalizations, and deaths. Foodborne nontyphoidal *Salmonella* strains were estimated to cause 1.03 million U.S. infections, 19,300 hospitalizations, and 378 deaths a year, resulting in an estimated $4.1 billion in annual direct medical costs, productivity loss, and premature death (Scallan et al., 2011; United States Department of Agriculture Economic Research Service, 2018). Despite efforts to reduce the incidence by 25% to meet the U.S. Department of Health and Human Services’ Healthy People goals for 2020 and 2030 (United States Department of Agriculture Food Safety and Inspection Service, 2020; US Department of Health and Human Services), incidence has remained relatively stable during 1996–2019 (decreases during 2020 and 2021 were likely related to the COVID-19 pandemic) (Centers for Disease Control and Prevention, 2021; Collins, 2022; Ray et al., 2021). Although more than 2,500 *Salmonella* serotypes have been described, the top 20 cause nearly 70% of U.S. *Salmonella* infections (Centers for Disease Control and Prevention, 2011; Issenhuth-Jeanjean et al., 2014). Some are commonly associated with specific foods (Foley et al., 2008; Jackson et al., 2013).

Although most *Salmonella* infections are sporadic (i.e., not linked to a recognized outbreak), outbreak data allow illnesses to be linked to specific food sources (Marshall et al., 2020). Data from outbreak investigations are used in a model to annually estimate the percentage of foodborne *Salmonella* illnesses that can be attributed to each food category (Batz et al., 2021). The model attributed 42% of *Salmonella* illnesses during 2020 to meat and poultry, including chicken (17%), pork (13%), beef (6%), and turkey (6%) (The Interagency Food Safety Analytics Collaboration, 2020). Meat and poultry have been implicated in several large multistate outbreaks in the last five years. In 2018, ground beef contaminated by serotype Newport caused 436 illnesses in an outbreak that was the largest *Salmonella* outbreak associated with ground beef since 1998, resulting in a recall of 12 million pounds of ground beef (Centers for Disease Control and Prevention, 2018). During 2017–2019, an emerging *Salmonella* Reading strain resulted in 356 illnesses associated with various turkey products, leading to recalls of 3,000 lb of ground turkey products made for human consumption and raw turkey pet food (Hassan et al., 2019).

Determining which serotypes are causing the most outbreak illnesses transmitted by each meat or poultry type and which serotypes may be emerging as major causes of outbreak illnesses can inform serotype-specific public health interventions. To complement models and other data sources (Batz et al., 2021; The Interagency Food Safety Analytics Collaboration, 2020), we developed a rapid, simple approach to classify *Salmonella* outbreak data by burden and trajectory. For each meat and poultry type, we (1) determined which serotypes were major causes of recent outbreak illnesses (i.e., outbreak illness burden), (2) identified serotypes that have recently increased, decreased, or remained stable as a cause of illness (i.e., outbreak illness trajectory), (3) identified serotypes that recently emerged as causes of outbreak illnesses. To display these data and facilitate accessibility, visualization, and use, we created a dashboard using this approach for public use (https://www.cdc.gov/ncezid/dfwed/BEAM-dash-board.html). This approach, along with data from sporadic infections in humans, data from animals, and meat and poultry product testing data, can help identify serotypes for prevention efforts.

## Methods

We examined reports of foodborne *Salmonella* outbreaks with chicken, turkey, beef, or pork listed as the confirmed or suspected source during 2012–2021 using data from CDC’s Foodborne Disease Outbreak Surveillance System (FDOSS). We defined a foodborne outbreak as an incident in which two or more persons experience a similar illness after ingestion of a common food, and epidemiologic analysis implicated food as the source (Centers for Disease Control and Prevention, 2017). We included only outbreaks with *Salmonella* as the confirmed etiology that were assigned to a single food type of chicken, turkey, beef, or pork using the Interagency Food Safety Analytics Collaboration categorization scheme (Richardson et al., 2017). We included outbreaks with chicken, turkey, beef, or pork as the confirmed or suspected food vehicle. We did not include outbreaks linked to a multiingredient food if the single responsible food type was not determined. We excluded outbreaks caused by multiple serotypes.

We examined the total and annual number of foodborne *Salmonella* outbreaks and outbreak illnesses, hospitalizations, and deaths during 2012–2021, by serotype, for each of the four major meat and poultry categories. We assigned each outbreak to the year it started, which was determined using the date of first illness onset. For each serotype associated with a meat or poultry outbreak during the most recent 5 years (2017–2021), we determined the total number of meat or poultry outbreak illnesses during those years. We analyzed the number of outbreak illnesses, not the number of outbreaks, for three reasons: (1) we aim to reduce or prevent illness, therefore analyzing outbreak illnesses aligns more closely with this goal; (2) outbreaks may represent at least four different scenarios, including contamination at: a single retail location or event, a processing facility resulting in either a small or large amount of contaminated product, a farm that results in contaminated product at several processing facilities, several farms or is widespread throughout the industry resulting in contamination across many processing facilities. Outbreak investigations rarely determine which scenario has occurred because data needed to determine this are often unavailable. The number of outbreak illnesses can provide some information on the scope of the contamination event; (3) the number of outbreaks, even combined over a 5-year period, is still relatively small making analyses less stable.

We defined burden as the total number of meat or poultry outbreak illnesses during the most recent 5 years. We determined the burden category (high, moderate, low) of individual serotypes for each meat and poultry type as follows: high (75th percentile of serotype-specific total outbreak illnesses during 2017–2021), moderate (51–74th percentile), or low (50th percentile and below). We defined trajectory as the relative change in the total number of meat or poultry outbreak illnesses for individual serotypes from the previous five years (2012–2016) to the most recent five years (2017–2021), by meat and poultry type. We classified the trajectory as increased, stable, or decreased. We defined increased trajectory as an increase in illnesses of 50% or more; stable as an increase or decrease of less than 50%; and decreased trajectory as a decrease of 50% or more. We did not conduct analyses to examine whether changes in the number of illnesses between the two 5-year periods were statistically significant. We defined a serotype as recently emerged for a given meat or poultry type if it caused outbreak illnesses during the most recent five years (2017–2021) but not during 1998–2011. We chose 1998 partly because of changes in data collection and reporting in 1998.

## Results

During 2012–2021, CDC received reports of 192 *Salmonella* outbreaks associated with the consumption of chicken, turkey, beef, or pork, resulting in 7,077 illnesses, 1,330 hospitalizations, and 9 deaths (Supplemental Table). Of these, 88 (46%) outbreaks with 2,935 (41%) illnesses were associated with chicken, 47 (24%) outbreaks with 1,699 (24%) illnesses were associated with pork, 33 (17%) outbreaks with 1,255 (18%) illnesses were associated with beef, and 24 (13%) outbreaks with 1,188 (17%) illnesses were associated with turkey (Table 1). Outbreaks were caused by 34 *Salmonella* serotypes. Seven serotypes (Braenderup, Enteritidis, I 4,[5],12:i:-, Javiana, Muenchen, Newport, and Typhimurium) caused outbreaks associated with all four meat and poultry types. Two serotypes caused outbreaks associated with three meat and poultry types (Infantis, Schwarzengrund). Five serotypes caused outbreaks in two types: Anatum and Saintpaul in chicken and turkey, Heidelberg in chicken and beef, Thompson in chicken and pork, and Uganda in beef and pork. Twenty serotypes caused outbreaks in only one type. The outbreak illness burden and trajectory for each serotype for each food type are provided in Table 1. In the paragraphs below, we provide names of serotypes with high burden and any trajectory or with moderate burden and increased trajectory. We highlight some serotypes of concern based on a combination of outbreak illness burden and trajectory.

### *Salmonella* outbreaks associated with chicken

**Figure 1.**
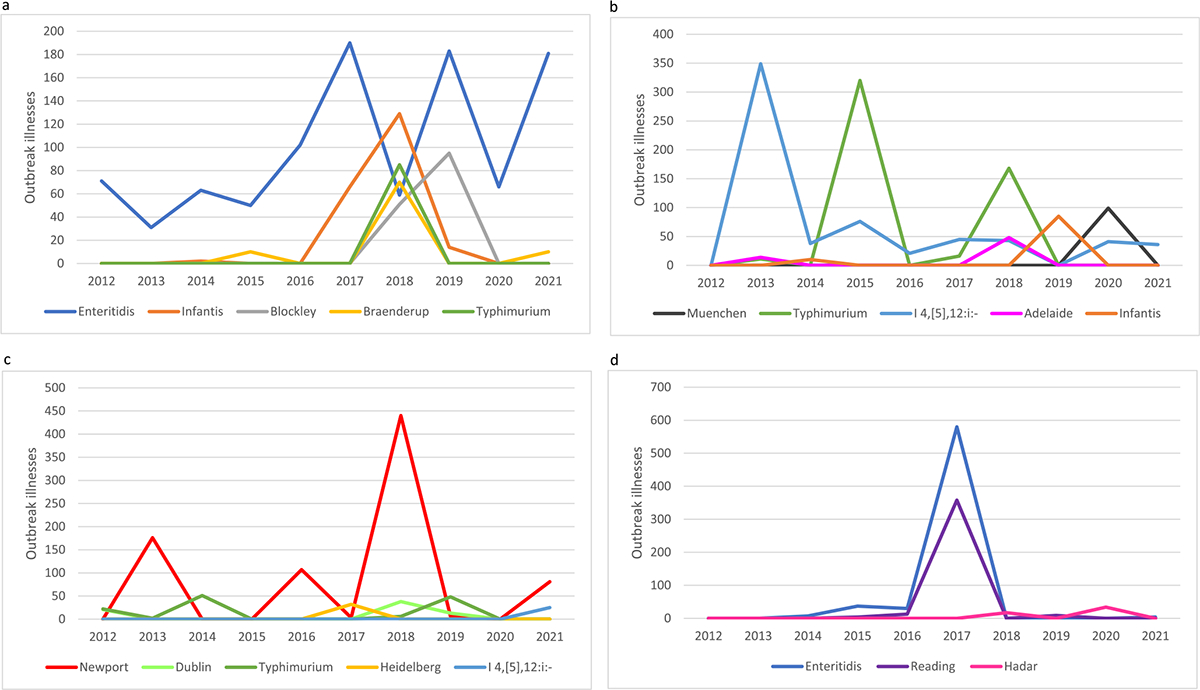
Annual number of foodborne outbreak illnesses associated with (a) chicken, (b) pork, (c) beef, and (d) turkey, by serotype, for serotypes that were categorized as high burden and increased, stable or decreased trajectory, or moderate burden and increased trajectory, 2012–2021.

Eighty-eight *Salmonella* outbreaks with 2,935 illnesses, 611 hospitalizations, and 4 deaths were associated with chicken during 2012–2021. Forty-four outbreaks were reported in the most recent five years and 44 outbreaks were reported in the previous five years; the number of outbreak illnesses was lower (1,327 vs. 1,608) in the most recent five years (Table 1). Nineteen serotypes caused these 88 outbreaks; nine caused more than one outbreak. Of the 10 serotypes that caused outbreaks during the most recent 5 years, three were classified as high burden, two as moderate, and five as low burden (Table 2). Seven serotypes were classified as having an increased trajectory, one as stable, and two as decreased trajectory. Three serotypes were classified as high burden and increased trajectory (Enteritidis, Infantis, Blockley), none as high burden and stable or decreased trajectory, and two as moderate burden and increased trajectory (Braenderup, Typhimurium) (Fig. 1a). Two serotypes (Enteritidis and Heidelberg) caused outbreaks during ≥ 7 of the 10 years; one (Infantis) caused outbreaks during 4–6 of the years, and the remaining 16 serotypes caused outbreaks during ≤3 of the years. We identified two recently emerged serotypes, Anatum (2017) and Blockley (2018).

### *Salmonella* outbreaks associated with pork

Forty-seven outbreaks with 1,699 illnesses, 246 hospitalizations, and 2 deaths were associated with pork during 2012–2021. Fewer outbreaks (20 vs. 27) and outbreak-associated illnesses (666 vs. 1,033) occurred during 2017–2021 compared with 2012–2016 (Table 1). Nineteen serotypes caused these 47 outbreaks; nine caused more than one outbreak. Of the 10 serotypes that caused outbreaks during the most recent 5 years, three were classified as high burden, two as moderate burden, and five as low burden (Table 2). Eight serotypes were classified as having an increased trajectory, one as stable, and one as decreased trajectory. One serotype was classified as high burden and increased trajectory (Muenchen), one as high burden and stable trajectory (Typhimurium), one as high burden and decreased trajectory (I 4,[5],12:i:-), and two as moderate burden and increased trajectory (Adelaide, Infantis) (Fig. 1b). One serotype caused outbreaks associated with pork during ≥7 of the 10 years (I 4,[5],12:i:-); one serotype (Typhimurium) caused outbreaks during 4–6 of the years, and the remaining 17 serotypes caused outbreaks during ≤3 of the years. We identified three recently emerged serotypes, Eastbourne (2018), Muenchen (2020), and Schwarzengrund (2017).

### *Salmonella* outbreaks associated with beef

Thirty-three outbreaks with 1,255 illnesses, 296 hospitalizations, and 2 deaths were associated with beef during 2012–2021. More outbreaks (19 vs. 14) and outbreak illnesses (757 vs. 498) occurred during 2017–2021 compared with 2012–2016 (Table 1). Fourteen serotypes caused these 33 outbreaks; six caused more than one outbreak. Of the 11 serotypes that caused outbreaks during the most recent 5 years, three were classified as high burden, two as moderate burden, and six as low burden (Table 2). Eight serotypes were classified as having an increased trajectory, two as stable, and one as decreased trajectory. Two serotypes were classified as high burden and increased trajectory (Newport, Dublin), one as high burden and stable trajectory (Typhimurium), none as high burden and decreased trajectory, and two as moderate burden and increased trajectory (Heidelberg, I 4,[5],12:i:-) (Fig. 1c). No serotypes caused outbreaks associated with beef during ≥7 of the 10 years; two serotypes (Newport, Typhimurium) caused outbreaks during 4–6 of the years, and the remaining 12 serotypes caused outbreaks during ≤3 of the years. We identified two recently emerged serotypes, Braenderup (2017) and I 4,[5],12:i:- (2021).

### *Salmonella* outbreaks associated with turkey

Twenty-four outbreaks with 1,188 illnesses, 177 hospitalizations, and 1 death were associated with turkey during 2012–2021. There were fewer *Salmonella* outbreaks (11 vs. 13) but more outbreak-associated illnesses (1,029 vs. 159) during 2017–2021 compared with 2012–2016 (Table 1). Twelve serotypes caused these 24 outbreaks; five caused more than one outbreak. Of the seven serotypes that caused outbreaks during the most recent 5 years, two were classified as high burden, one as moderate burden, and four as low burden (Table 2). Six serotypes were classified as having an increased trajectory, none as stable, and one as decreased trajectory. Two serotypes were classified as high burden and increased trajectory (Enteritidis, Reading), none as high burden and stable or decreased trajectory, one as moderate burden and increased trajectory (Hadar) (Fig. 1d). No serotypes caused outbreaks associated with turkey during ≥7 of the 10 years; two (Enteritidis, Reading) caused outbreaks during 4–6 of the years, and the remaining 10 serotypes caused outbreaks during ≤3 of the years. We identified two recently emerged serotypes, Anatum (2019) and Schwarzengrund (2018).

## Discussion

We present a simple approach to describe and display outbreak data on *Salmonella* serotypes of concern that caused outbreak illnesses associated with consumption of each of four meat and poultry types. These calculations can be performed annually for the most recent 10-year period for which outbreak data are available (https://www.cdc.gov/ncezid/dfwed/BEAM-dashboard.html). Calculation and public display of outbreak illness burden and trajectory annually could help highlight outbreak serotypes that are of public health concern, and facilitate prioritization of serotypes for implementation of prevention measures by industry, federal and state regulatory, and health and agriculture agencies.

We identified serotypes of greatest concern for outbreaks as those with a high burden of outbreak illnesses that also have increased trajectories. These were Enteritidis (chicken and turkey), Blockley and Infantis (chicken), Reading (turkey), Dublin and Newport (beef), and Muenchen (pork). Serotypes with moderate outbreak illness burden and increased trajectory could become high burden; we also consider these of concern. One or two serotypes were in this category for each meat or poultry type. These were Braenderup and Typhimurium (chicken), Hadar (turkey), Heidelberg and I 4,[5],12:i:- (beef), and Adelaide and Infantis (pork). Serotypes with high burden and stable trajectory are also of concern. The only serotype in this category was Typhimurium (beef and pork). For these three burden/trajectory categories, federal and state regulatory, and health and agriculture partners should evaluate current prevention measures. Intensified prevention measures may be needed for serotypes with high or moderate burden and increased trajectory. Enhancements in prevention measures may be needed for serotypes with high burden and stable trajectory. Serotypes in any of these three categories for two or more meat and poultry types are of particular public health concern. These were Enteritidis for chicken and turkey; Infantis for pork and chicken; and Typhimurium for chicken, pork, and beef.

We identified nine serotypes that emerged during the most recent five years in a meat and poultry type: three in pork and two in each of the other food types. One of these (Anatum) emerged in both chicken and turkey. Information to be evaluated should include the timing of emergence and the number of meat and poultry types involved as well as the burden and trajectory categories. For example, Blockley outbreak illnesses due to chicken recently emerged (2018); the serotype is categorized as high burden and increased trajectory for chicken and was not reported among other meat and poultry types. It caused a U.S. outbreak associated with eggs in 1967 (Morse & Rubenstein, 1967) and has historically been associated with chicken in Southeast Asia and Europe (Bangtrakulnonth et al., 1994; Fell et al., 2000; Limawongpranee et al., 1999; Tassios et al., 2000; Threlfall et al., 2003; Wilson & Whitehead, 2006). The emergence of Blockley might represent an isolated event at one farm or processing facility or the beginning of wider transmission among chicken flocks in the United States. Evaluating concurrent data from animals and meat and poultry products, and subsequent years of outbreak and sporadic illness data could help in evaluating this emergence. Serotype I 4,[5],12:i:- recently emerged in beef (2021) and has been observed in pork (high burden, decreased trajectory), chicken (low burden, stable trajectory), and turkey (low burden, decreased trajectory). Antimicrobial−resistant, pork-associated infections caused by I 4,[5],12:i:- began increasing in Europe in the 1990s (Echeita et al., 1999; Moreno Switt et al., 2009). This strain emerged in swine and pork in the United States in the mid-2000s (Naberhaus et al., 2019). The first identified U.S. outbreak caused by highly resistant I 4,[5],12:i:- occurred in 2011 and was associated with pork (Self et al., 2017). The emergence in beef suggests that I 4,[5],12:i:- is now widespread among all meat and poultry food animals. For all serotypes, frequent review of surveillance data from humans and animals and from samples of meat and poultry products might help detect emerging serotypes earlier and determine the extent of their spread. The National Veterinary Services Laboratories culture clinical and nonclinical samples from many types of animals for *Salmonella* and publish reports of findings; isolations from animals could provide an early warning about serotypes that could emerge as a source of human illness (Morningstar-Shaw BR, 2016).

Detecting, characterizing, monitoring, and evaluating serotypes that emerge and cause illness associated with multiple meat and poultry types could offer an opportunity to examine how and why they emerged and even how pathogens move into food animals. Similarly, serotypes that were categorized as high burden and increased or stable trajectory or moderate burden and increased trajectory in multiple meat and poultry types warrant the exploration of possible common sources of transmission, including animal feed or animal feed ingredients (including feed derived from food animal sources), movement of food animals, wild animal sources, shared environment or water sources, and cross-contamination of foods. Understanding the factors that led to a serotype’s emergence and presence across multiple meat and poultry types could inform prevention measures.

Evaluating serotypes with decreased trajectory could help to identify effective prevention measures. The recent decline of outbreak-associated illnesses caused by Heidelberg associated with chicken (categorized as low burden and decreased trajectory) is likely related to prevention measures implemented by the chicken industry, including vaccinating chickens against Heidelberg and other interventions implemented after a large outbreak in 2013 linked to a single company (Gieraltowski et al., 2016). After the outbreak, the first regulatory performance standards for chicken parts were developed (New Performance Standards, 2016). Measures the company implemented include an environmental control program based on findings from environmental monitoring and testing for *Salmonella* at farms (breeder flocks, hatcheries, grow-out farms) and during production (processing establishments) that identified some farms as more likely to have *Salmonella*, changes to their operation and equipment, and vaccination of flocks (Charles, 2014; Gieraltowski et al., 2016). These measures reduced the prevalence of *Salmonella* on chicken and chicken parts to 5% or less at the implicated establishments (Charles, 2014; Gieraltowski et al., 2016). This suggests that targeted prevention measures can be effective in preventing illness and can catalyze improved standards, highlighting the critical and interconnected roles of regulators, industries, suppliers, retailers, consumers, and public health in addressing food safety.

Serotype data could inform existing prevention measures used by food producers and regulatory agencies to help decrease illness caused by *Salmonella* (Gast, 2007). Many chicken and egg producers vaccinate chickens against certain *Salmonella* serotypes using commercially developed or autogenous vaccines (Dórea et al., 2010; USDA, 2014, 2011); vaccination has contributed to the decline of *Salmonella* Enteritidis in the UK (O’Brien, 2012). Regulatory authorities have also developed guidelines and plans that include serotype-specific strategies, including USDA FSIS’s proposed regulatory framework to reduce *Salmonella* illnesses attributable to poultry (USDA Food Safety and Inspection Service, 2022). Animal feed can be a source of human illness attributed to the consumption of meat or poultry, but routine feed testing is very limited (Jones, 2011; Reiter et al., 2012). Testing purchased animal feed for *Salmonella* is included in the industry-developed voluntary Beef Quality Assurance plan (Beef Quality Assurance, 2019). However, the FDA only considers animal feed adulterated if it is contaminated with a *Salmonella* serotype that is also pathogenic to the animal consuming the feed (e.g., Enteritidis for poultry, Newport and Dublin for cattle) (Center for Veterinary Medicine 2013). Monitoring of poultry flocks for *Salmonella* Enteritidis is included in the voluntary National Poultry Improvement Plan (7 U.S.C. 8301–8317). Other measures that could be considered include the development of retailer purchase specifications that include prevention measures when specific serotypes, or serotypes that generally cause illness, are detected at poultry suppliers (Walmart, 2017). Local, state, and federal public health, regulatory, and agriculture agencies could prioritize investigations of outbreaks caused by these serotypes; traceback investigations and information provided by farms could help inform the development of prevention strategies. Serotype-specific measures that have been used to reduce other pathogens could also be considered for *Salmonella*. After several large outbreaks in the 1990s, USDA FSIS declared STEC O157 an adulterant in ground beef and later added six non-O157 STEC serotypes (Wheeler et al., 2014). Currently, *Salmonella* is not considered an adulterant in not-ready-to-eat meat and poultry products. However, USDA FSIS, as part of the proposed regulatory framework to reduce *Salmonella* illnesses attributable to poultry, is assessing whether certain types of *Salmonella* or contamination levels should be considered adulterants in raw poultry sold to consumers (USDA Food Safety and Inspection Service, 2022).

These findings are subject to several limitations. First, only about 9% of reported *Salmonella* illnesses are associated with outbreaks and outbreaks might not represent the major serotypes responsible for all illnesses due to a particular food (Ray et al., 2021). Second, outbreaks in this analysis may be an underestimate or may not be representative of all *Salmonella* outbreaks associated with chicken, turkey, beef, and pork. Not all *Salmonella* outbreaks are captured in FDOSS because reporting by local and state health departments is voluntary. For many outbreaks, the food source is never identified (Centers for Disease Control and Prevention, 2019; Marshall et al., 2020). Third, our analysis did not include outbreaks associated with foods that contain multiple ingredients if the responsible ingredient was not determined. Nearly 50% of outbreaks are linked to foods that have multiple ingredients; chicken, other poultry, and meats are consumed frequently and can be ingredients in many dishes (Centers for Disease Control and Prevention, 2019; Marshall et al., 2020). Fourth, the number of illnesses linked to each outbreak is likely underestimated because many persons with diarrheal illness do not seek medical care or have a stool specimen tested (Scallan et al., 2011); the number might vary by type and location of outbreak, serotype, severity of illness, or other factors. During 2020 and 2021, fewer outbreaks and illnesses were reported to CDC due to the COVID-19 pandemic, likely due to both decreased occurrence and fewer investigations (Collins, 2022; Ray et al., 2021). This could affect the burden and trajectory calculations. Fifth, because the unit of analysis was outbreak illnesses, the importance of outbreaks with many illnesses may be overweighted. We chose to keep the analyses simple rather than to create a model to account for this and other factors. In the future, it may be possible to use whole genome sequencing to develop hypotheses about likely food sources. Lastly, the high, moderate, and low categories for each serotype-food pair are determined using percentiles calculated for a given 5-year period; they are relative terms for outbreak illness burden. A high burden of illness for one serotype-food pair during a particular five-year period may be a similar number of illnesses that is characterized as moderate or low burden for another serotype-food pair.

Characterizing the outbreak illness burden and trajectory for serotypes transmitted by these foods using the latest available data can help in identifying important and emerging *Salmonella* serotype-food pairs. More measures should be implemented to reduce illnesses associated with *Salmonella* serotypes of public health concern. Though not serotype-specific, improving hygiene measures along the food production chain, including on the farm, at slaughter, during food production, and preparation could also help prevent illness. All *Salmonella* serotypes, regardless of outbreak illness burden and trajectory, should continue to be monitored through surveillance. This includes surveillance for sporadic illnesses and outbreaks in people, carriage by chicken, turkey, cattle, and swine, and contamination of meat and poultry products made from these animals. Partnerships between public health agencies, regulatory agencies, industry, academia, and consumer groups can help in identifying, developing, refining, and evaluating serotype-specific and general prevention measures to reduce foodborne illness.

## Supplementary Material

An Approach to Describe Salmonella Serotypes of Concern for Outbreaks_Supplemental table

## Figures and Tables

**Table 1:** Number of outbreaks and outbreak illnesses, by meat or poultry type and serotype, 2012–2016 and 2017–2021, with calculated outbreak illness burden and trajectory

Food type	Serotype	2012–2016		2017–2021		Total		Outbreak illness burden, 2017–2021^†^	Outbreak illness trajectory^‡^
		Outbreaks No.	Outbreak illnesses No.	Outbreaks No.	Outbreak illnesses No.	Outbreaks No.	Outbreak illnesses No.		
**Meat or poultry**	**All**	**98**	**3298**	**94**	**3779**	**192**	**7077**	–	–
**Chicken**	**All**	**44**	**1608**	**44**	**1327**	**88**	**2935**	–	–
	Enteritidis	19	317	27	679	46	996	High	Increased
	Infantis	1	2	3	209	4	211	High	Increased
	Blockley*	0	0	2	146	2	146	High	Increased
	Braenderup	1	10	2	80	3	90	Moderate	Increased
	Typhimurium	0	0	1	85	1	85	Moderate	Increased
	Paratyphi B	0	0	1	7	1	7	Low	Increased
	Anatum*	0	0	1	4	1	4	Low	Increased
	I 4,[5],12:i:-	1	64	3	39	4	103	Low	Stable
	Heidelberg	8	880	3	63	11	943	Low	Decreased
	Thompson	3	140	1	15	4	155	Low	Decreased
	Carmel	1	33	0	0	1	33	No outbreak illnesses^§^	Decreased
	Derby	1	3	0	0	1	3	No outbreak illnesses^§^	Decreased
	Javiana	2	45	0	0	2	45	No outbreak illnesses^§^	Decreased
	Montevideo	1	5	0	0	1	5	No outbreak illnesses^§^	Decreased
	Muenchen	2	5	0	0	2	5	No outbreak illnesses^§^	Decreased
	Newport	1	8	0	0	1	8	No outbreak illnesses^§^	Decreased
	Norwich	1	10	0	0	1	10	No outbreak illnesses^§^	Decreased
	Saintpaul	1	70	0	0	1	70	No outbreak illnesses^§^	Decreased
	Schwarzengrund	1	16	0	0	1	16	No outbreak illnesses^§^	Decreased
**Pork**	**All**	**27**	**1033**	**20**	**666**	**47**	**1699**	–	–
	Muenchen*	0	0	1	99	1	99	High	Increased
	Typhimurium	4	331	5	184	9	515	High	Stable
	I 4,[5],12:i:-	8	484	7	165	15	649	High	Decreased
	Adelaide	1	14	2	48	3	62	Moderate	Increased
	Infantis	1	10	1	85	2	95	Moderate	Increased
	Berta	0	0	1	30	1	30	Low	Increased
	Eastbourne*	0	0	1	21	1	21	Low	Increased
	Schwarzengrund*	0	0	1	30	1	30	Low	Increased
	Muenster	0	0	1	4	1	4	Low	Increased
	Agona	2	15	0	0	2	15	No outbreak illnesses^§^	Decreased
	Braenderup	1	8	0	0	1	8	No outbreak illnesses^§^	Decreased
	Enteritidis	2	41	0	0	2	41	No outbreak illnesses^§^	Decreased
	Goldcoast	1	12	0	0	1	12	No outbreak illnesses^§^	Decreased
	Javiana	1	41	0	0	1	41	No outbreak illnesses^§^	Decreased
	Mbandaka	2	27	0	0	2	27	No outbreak illnesses^§^	Decreased
	Newport	1	20	0	0	1	20	No outbreak illnesses^§^	Decreased
	Thompson	1	11	0	0	1	11	No outbreak illnesses^§^	Decreased
	Uganda	2	19	0	0	2	19	No outbreak illnesses^§^	Decreased
**Beef**	**All**	**14**	**498**	**19**	**757**	**33**	**1255**	–	–
	Dublin	1	21	2	51	3	72	High	Increased
	Newport	3	283	6	530	9	813	High	Increased
	Typhimurium	4	75	2	54	6	129	High	Stable
	Heidelberg	0	0	1	32	1	32	Moderate	Increased
	I 4,[5],12:i:-*	0	0	1	25	1	25	Moderate	Increased
	Oranienburg	0	0	1	18	1	18	Low	Increased
	Braenderup*	0	0	2	11	2	11	Low	Increased
	Infantis	0	0	1	4	1	4	Low	Increased
	Kiambu	0	0	1	8	1	8	Low	Increased
	Uganda	1	25	1	18	2	43	Low	Stable
	Enteritidis	2	75	1	6	3	81	Low	Decreased
	Javiana	1	7	0	0	1	7	No outbreak illnesses^§^	Decreased
	Muenchen	1	3	0	0	1	3	No outbreak illnesses^§^	Decreased
	Potsdam	1	9	0	0	1	9	No outbreak illnesses^§^	Decreased
**Turkey**	**All**	**13**	**159**	**11**	**1029**	**24**	**1188**	–	–
	Enteritidis	4	74	3	584	7	658	High	Increased
	Reading	2	17	2	367	4	384	High	Increased
	Hadar	0	0	2	51	2	51	Moderate	Increased
	Anatum*	0	0	1	8	1	8	Low	Increased
	Newport	0	0	1	9	1	9	Low	Increased
	Schwarzengrund*	0	0	1	7	1	7	Low	Increased
	I 4,[5],12:i:-	1	10	1	3	2	13	Low	Decreased
	Braenderup	1	4	0	0	1	4	No outbreak illnesses^§^	Decreased
	Javiana	1	20	0	0	1	20	No outbreak illnesses^§^	Decreased
	Muenchen	1	14	0	0	1	14	No outbreak illnesses^§^	Decreased
	Saintpaul	2	13	0	0	2	13	No outbreak illnesses^§^	Decreased
	Typhimurium	1	7	0	0	1	7	No outbreak illnesses^§^	Decreased

* Recently emerged. These are serotypes that caused an outbreak associated with a particular meat or poultry type during 2017–2021 but did not cause an outbreak associated with that type during 1998–2011.

† Determined using outbreak illnesses during 2017–2021 for individual serotypes that caused at least one outbreak in a food type. High indicates serotypes with outbreak illnesses in the ≥75th percentile, moderate with outbreak illnesses in the 51st–74th percentile, and low with outbreak illnesses in the 50th percentile and below.

‡ Determined by comparing outbreak illnesses during 2017–2021 with 2012–2016. Increased indicates serotypes with an increase in illnesses of 50% or more; stable an increase or decrease of less than 50%; and decreased a decrease of 50% or more illnesses.

§ Serotype that did not cause an outbreak during the most recent 5 years but caused an outbreak during the previous 5 years.

**Table 2:** Outbreak illness burden, trajectory, and corresponding *Salmonella* serotypes associated with consumption of chicken, turkey, beef, and pork during 2017–2021 compared with 2012–2016, and possible approaches to reduce illness

		*Salmonella* serotypes				
Outbreak illness burden (recent 5 years, 2017–2021)	Outbreak illness trajectory (recent 5 years vs. previous 5 years)	Chicken	Turkey	Beef	Pork	Possible approaches to reduce illness**
High	Increased	Enteritidis, Infantis, Blockley*	Enteritidis, Reading	Dublin, Newport	Muenchen*	Intensify prevention measures
High	Stable	—	—	Typhimurium	Typhimurium	Enhance prevention measures
High	Decreased	—	—	—	I 4,[5],12:i:-	Continue prevention measures
Moderate	Increased	Braenderup, Typhimurium	Hadar	Heidelberg, I 4,[5],12:i:-*	Adelaide, Infantis	Intensify prevention measures
Moderate	Stable	—	—	—	—	Continue prevention measures
Moderate	Decreased	—	—	—	—	Continue prevention measures
Low	Increased	Anatum*, Paratyphi B	Anatum*, Newport, Schwarzengrund*	Braenderup*, Infantis, Kiambu, Oranienburg	Berta, Eastbourne*, Muenster, Schwarzengrund*	Continue prevention measures
Low	Stable	I 4,[5],12:i:-	—	Uganda	—	Continue prevention measures
Low	Decreased	Heidelberg, Thompson	I 4,[5],12:i:-	Enteritidis		Continue prevention measures
No outbreak illnesses	Decreased	Carmel, Derby, Javiana, Montevideo, Muenchen Newport, Norwich, Saintpaul, Schwarzengrund	Braenderup, Javiana, Muenchen, Saintpaul, Typhimurium	Javiana, Muenchen, Potsdam	Agona, Braenderup, Enteritidis, Goldcoast, Javiana, Mbandaka, Newport, Thompson, Uganda	Continue prevention measures

† Determined using outbreak illnesses during 2017–2021 for individual serotypes that caused at least one outbreak in a food type. High indicates serotypes with outbreak illnesses in the ≥75th percentile, moderate to 51st–74th percentile, and low to 50th percentile and below.

‡ Determined by comparing outbreak illnesses during 2017–2021 with 2012–2016. Increased indicates serotypes with an increase in illnesses of 50% or more; stable an increase or decrease of less than 50%; and decreased a decrease of 50% or more illnesses.

* Recently emerged. These are serotypes that caused an outbreak associated with a particular meat or poultry type during 2017–2021 but did not cause an outbreak associated with that type during 1998–2011.

** The approach should be determined after considering other data sources, e.g., human sporadic illnesses, isolations from animal and foods.
