# Supplementary material for: An Approach to Describe Salmonella Serotypes of Concern for Outbreaks: Using Burden and Trajectory of Outbreak-related Illnesses Associated with Meat and Poultry
Source: J Food Prot. Author manuscript; Available in PMC 2024 Sep 2. (PMC11366486; doi:10.1016/j.jfp.2024.100331)
Supplement: An Approach to Describe Salmonella Serotypes of Concern for Outbreaks_Supplemental table [file NIHMS2015315-supplement-An_Approach_to_Describe_Salmonella_Serotypes_of_Concern_for_Outbreaks_Supplemental_table.docx]

**Supplemental Table.** Foodborne salmonella outbreaks associated with chicken, pork, beef, and turkey, 2012-2021 (n=192)*.

| **Year** | **Serotype** | **Outbreak Illnesses No.** | **Hospitalizations No.** | **Deaths No.** | **Single State or Multistate** | **Food type** | **Food name** |
| --- | --- | --- | --- | --- | --- | --- | --- |
| 2012 | Newport | 8 | 3 | 0 | Single state | Chicken | chicken |
| 2012 | Enteritidis | 38 | 1 | 0 | Single state | Chicken | fajita, chicken |
| 2012 | Dublin | 21 | 5 | 0 | Single state | Beef | beef, laab raw |
| 2012 | Heidelberg | 134 | 33 | 0 | Multistate | Chicken | chicken |
| 2012 | Enteritidis | 51 | NA | 0 | Multistate | Beef | ground beef |
| 2012 | Schwarzengrund | 16 | NA | NA | Multistate | Chicken | chicken |
| 2012 | Enteritidis | 24 | 2 | 0 | Single state | Beef | beef, raw |
| 2012 | Uganda | 7 | 5 | 0 | Single state | Pork | Hog Head Cheese |
| 2012 | Enteritidis | 33 | 1 | 0 | Single state | Chicken | chicken |
| 2012 | Javiana | 43 | 40 | 0 | Single state | Chicken | chicken, baked |
| 2012 | Saintpaul | 2 | 0 | 0 | Single state | Turkey | turkey, smoked |
| 2012 | Typhimurium | 7 | 3 | 0 | Single state | Turkey | turkey |
| 2012 | Typhimurium | 22 | 7 | 0 | Multistate | Beef | ground beef |
| 2013 | Montevideo | 5 | 0 | 0 | Single state | Chicken | chicken, BBQ |
| 2013 | Heidelberg | 634 | 200 | 0 | Multistate | Chicken | chicken |
| 2013 | Heidelberg | 17 | 5 | 0 | Single state | Chicken | chicken |
| 2013 | Enteritidis | 35 | 1 | 0 | Single state | Pork | ham |
| 2013 | Newport | 39 | 9 | 0 | Multistate | Beef | ground beef |
| 2013 | I 4,[5],12:i:- | 333 | 50 | 0 | Single state | Pork | sausage, pork |
| 2013 | Newport | 137 | 6 | 0 | Single state | Beef | beef, raw and/or undercooked |
| 2013 | Uganda | 25 | 2 | 0 | Single state | Beef | beef, laab raw |
| 2013 | Enteritidis | 6 | 4 | 0 | Single state | Pork | pork, smoked |
| 2013 | Thompson | 11 | 4 | 0 | Single state | Pork | ribs, pork |
| 2013 | I 4,[5],12:i:- | 16 | 3 | 0 | Single state | Pork | pork, other |
| 2013 | Adelaide | 14 | 2 | 0 | Multistate | Pork | pork |
| 2013 | Javiana | 2 | 1 | 0 | Single state | Chicken | chicken |
| 2013 | Javiana | 7 | 0 | 0 | Single state | Beef | fajita, beef |
| 2013 | Enteritidis | 24 | 1 | 0 | Single state | Chicken | Ground Chicken |
| 2013 | Mbandaka | 10 | 0 | 0 | Single state | Pork | pork, roasted |
| 2013 | Typhimurium | 11 | 1 | 0 | Single state | Pork | pork, BBQ |
| 2013 | Muenchen | 14 | 1 | 0 | Single state | Turkey | turkey |
| 2013 | Braenderup | 4 | 0 | 0 | Single state | Turkey | turkey leg |
| 2013 | Enteritidis | 3 | 0 | 0 | Single state | Chicken | chicken |
| 2013 | Enteritidis | 4 | 0 | 0 | Single state | Chicken | chicken |
| 2013 | Heidelberg | 7 | 3 | 0 | Single state | Chicken | chicken mole |
| 2013 | Saintpaul | 11 | 4 | 0 | Single state | Turkey | turkey |
| 2013 | Heidelberg | 24 | 3 | 0 | Single state | Chicken | chicken |
| 2013 | Typhimurium | 2 | 2 | 0 | Single state | Beef | steak |
| 2014 | Enteritidis | 20 | 0 | 0 | Single state | Chicken | chicken, casserole |
| 2014 | Infantis | 10 | 5 | 0 | Single state | Pork | pork |
| 2014 | Agona | 5 | 0 | 0 | Single state | Pork | pork |
| 2014 | Enteritidis | 18 | 1 | 0 | Single state | Chicken | chicken |
| 2014 | Heidelberg | 19 | 1 | 0 | Single state | Chicken | chicken, smoked |
| 2014 | Enteritidis | 8 | 3 | 0 | Single state | Chicken | chicken |
| 2014 | Typhimurium | 4 | 0 | 0 | Single state | Beef | ground beef, hamburger |
| 2014 | Enteritidis | 4 | 4 | 0 | Single state | Chicken | chicken |
| 2014 | I 4,[5],12:i:- | 18 | 2 | 0 | Single state | Pork | pork, roasted |
| 2014 | I 4,[5],12:i:- | 20 | 2 | 0 | Single state | Pork | pork, roasted |
| 2014 | Typhimurium | 47 | 11 | 0 | Multistate | Beef | ground beef |
| 2014 | Javiana | 20 | 1 | 0 | Single state | Turkey | turkey, roasted |
| 2014 | Braenderup | 8 | 2 | 0 | Single state | Pork | pork, carnitas |
| 2014 | Enteritidis | 5 | 0 | 0 | Single state | Chicken | chicken liver pate |
| 2014 | Enteritidis | 8 | 1 | 0 | Single state | Chicken | chicken, kiev |
| 2014 | Carmel | 33 | 1 | 0 | Single state | Chicken | chicken |
| 2014 | Thompson | 37 | 3 | 0 | Single state | Chicken | sandwich, chicken |
| 2014 | Enteritidis | 7 | 2 | 0 | Single state | Turkey | turkey |
| 2014 | Heidelberg | 36 | 5 | 0 | Single state | Chicken | chicken, grilled |
| 2014 | Infantis | 2 | 1 | 0 | Single state | Chicken | chicken |
| 2014 | Uganda | 12 | 2 | 0 | Single state | Pork | blood sausage |
| 2014 | Thompson | 56 | 4 | 0 | Single state | Chicken | chicken, smoked |
| 2015 | Enteritidis | 28 | NA | 0 | Single state | Turkey | Turkey, ground |
| 2015 | Enteritidis | 15 | 4 | 0 | Multistate | Chicken | stuffed chicken |
| 2015 | Enteritidis | 7 | 1 | 0 | Single state | Chicken | Chicken |
| 2015 | Enteritidis | 9 | 0 | 0 | Single state | Turkey | turkey |
| 2015 | I 4,[5],12:i:- | 73 | 7 | 0 | Single state | Pork | pork, carnitas |
| 2015 | Enteritidis | 5 | 2 | 0 | Single state | Chicken | stuffed chicken |
| 2015 | Typhimurium | 283 | 27 | 1 | Single state | Pork | pork, BBQ |
| 2015 | Agona | 10 | 0 | 0 | Single state | Pork | pork |
| 2015 | I 4,[5],12:i:- | 3 | 0 | 0 | Single state | Pork | pork |
| 2015 | Enteritidis | 10 | 1 | 0 | Single state | Chicken | Chicken Breast |
| 2015 | Enteritidis | 3 | 1 | 0 | Single state | Chicken | chicken tenders |
| 2015 | Derby | 3 | 0 | 0 | Single state | Chicken | chicken, rotisserie |
| 2015 | Muenchen | 3 | 0 | 0 | Single state | Chicken | Chicken Katsu Plate |
| 2015 | Braenderup | 10 | 0 | 0 | Single state | Chicken | chicken, rotisserie |
| 2015 | Typhimurium | 20 | 0 | 0 | Single state | Pork | pork, roasted |
| 2015 | Typhimurium | 17 | 6 | 0 | Single state | Pork | pork |
| 2015 | Enteritidis | 10 | 1 | 0 | Single state | Chicken | chicken, grilled |
| 2015 | I 4,[5],12:i:- | 10 | 0 | 0 | Single state | Turkey | turkey |
| 2015 | Mbandaka | 17 | 0 | 0 | Single state | Pork | pork, carnitas |
| 2015 | Reading | 4 | 0 | 0 | Single state | Turkey | turkey |
| 2015 | Potsdam | 9 | 1 | 0 | Single state | Beef | ox tongue & tripe |
| 2016 | Heidelberg | 9 | 1 | 0 | Single state | Chicken | chicken |
| 2016 | Goldcoast | 12 | 1 | 0 | Multistate | Pork | pork |
| 2016 | Enteritidis | 9 | 0 | 0 | Single state | Chicken | pate, chicken liver |
| 2016 | Enteritidis | 93 | 2 | 0 | Single state | Chicken | chicken, baked |
| 2016 | Enteritidis | 30 | 3 | 0 | Single state | Turkey | Turkey, ground |
| 2016 | Norwich | 10 | 1 | 0 | Single state | Chicken | chicken |
| 2016 | I 4,[5],12:i:- | 15 | 0 | 0 | Single state | Pork | pork, roasted |
| 2016 | I 4,[5],12:i:- | 64 | 16 | 0 | Multistate | Chicken | chicken |
| 2016 | Saintpaul | 70 | 12 | 0 | Multistate | Chicken | chicken |
| 2016 | I 4,[5],12:i:- | 6 | 3 | 0 | Single state | Pork | pork |
| 2016 | Muenchen | 2 | 2 | 1 | Single state | Chicken | chicken |
| 2016 | Muenchen | 3 | 1 | 0 | Single state | Beef | beef, brisket |
| 2016 | Thompson | 47 | 6 | 0 | Single state | Chicken | chicken |
| 2016 | Newport | 20 | 4 | 0 | Single state | Pork | smoked whole hog |
| 2016 | Javiana | 41 | 4 | 0 | Single state | Pork | pork, BBQ |
| 2016 | Newport | 107 | 43 | 1 | Multistate | Beef | ground beef |
| 2016 | Reading | 13 | 0 | 0 | Single state | Turkey | turkey |
| 2017 | Enteritidis | 6 | 1 | 0 | Single state | Beef | kitfo |
| 2017 | Enteritidis | 8 | 0 | 0 | Single state | Chicken | chicken |
| 2017 | Infantis | 66 | 0 | 0 | Single state | Chicken | chicken, smoked |
| 2017 | I 4,[5],12:i:- | 6 | 4 | 0 | Multistate | Chicken | chicken |
| 2017 | Enteritidis | 23 | 5 | 0 | Single state | Turkey | ground turkey, unspecified |
| 2017 | Braenderup | 4 | 2 | 0 | Single state | Beef | steak |
| 2017 | Enteritidis | 5 | 2 | 0 | Single state | Chicken | chicken |
| 2017 | Enteritidis | 104 | 0 | 0 | Single state | Chicken | chicken |
| 2017 | Enteritidis | 44 | 0 | 0 | Single state | Chicken | chicken |
| 2017 | Enteritidis | 3 | 0 | 0 | Single state | Chicken | chicken |
| 2017 | I 4,[5],12:i:- | 5 | 0 | 0 | Single state | Pork | whole pig |
| 2017 | Schwarzengrund | 30 | 11 | 1 | Single state | Pork | pork rib tips |
| 2017 | Typhimurium | 10 | 1 | 0 | Single state | Pork | Pulled Pork |
| 2017 | Enteritidis | 8 | 0 | 0 | Single state | Chicken | chicken salad sandwich |
| 2017 | Heidelberg | 32 | 6 | 0 | Single state | Beef | roast beef |
| 2017 | I 4,[5],12:i:- | 36 | 3 | 0 | Single state | Pork | pork |
| 2017 | Typhimurium | 6 | 1 | 0 | Single state | Pork | burrito, pork |
| 2017 | Braenderup | 7 | 1 | 0 | Single state | Beef | ribs, beef |
| 2017 | I 4,[5],12:i:- | 4 | 0 | 0 | Single state | Pork | whole pig |
| 2017 | I 4,[5],12:i:- | 25 | 11 | 1 | Multistate | Chicken | chicken |
| 2017 | Heidelberg | 33 | 9 | 0 | Single state | Chicken | chicken, pulled |
| 2017 | Enteritidis | 18 | 1 | 0 | Single state | Chicken | kabobs, chicken |
| 2017 | Enteritidis | 557 | 7 | 0 | Single state | Turkey | turkey |
| 2017 | Reading | 358 | 133 | 1 | Multistate | Turkey | turkey |
| 2017 | Anatum | 4 | 0 | 0 | Single state | Chicken | sandwich, chicken |
| 2017 | Newport | 3 | 2 | 0 | Single state | Beef | ground beef, raw |
| 2018 | Infantis | 129 | 25 | 1 | Multistate | Chicken | chicken |
| 2018 | Adelaide | 19 | 1 | 0 | Multistate | Pork | pork |
| 2018 | Enteritidis | 2 | 2 | 0 | Single state | Chicken | chicken |
| 2018 | Enteritidis | 16 | 3 | 0 | Single state | Chicken | smoked chicken |
| 2018 | I 4,[5],12:i:- | 8 | 1 | 0 | Single state | Chicken | pate, chicken liver |
| 2018 | Enteritidis | 9 | 3 | 0 | Single state | Chicken | chicken, other |
| 2018 | Enteritidis | 13 | 8 | 0 | Multistate | Chicken | Chicken, Frozen breaded |
| 2018 | Typhimurium | 85 | 22 | 0 | Multistate | Chicken | chicken |
| 2018 | Newport | 4 | 0 | 0 | Single state | Beef | beef |
| 2018 | Blockley | 51 | 7 | 0 | Multistate | Chicken | chicken, raw |
| 2018 | Heidelberg | 2 | 0 | 0 | Single state | Chicken | chicken, raw |
| 2018 | Infantis | 4 | 1 | 0 | Single state | Beef | Pamillo (Rib Steak) |
| 2018 | Typhimurium | 109 | 11 | 0 | Single state | Pork | Pulled Pork |
| 2018 | Dublin | 38 | 7 | 0 | Single state | Beef | beef |
| 2018 | Eastbourne | 21 | 7 | 0 | Multistate | Pork | pork |
| 2018 | Braenderup | 70 | 16 | 0 | Single state | Chicken | chicken, grilled |
| 2018 | Newport | 436 | 124 | 0 | Multistate | Beef | ground beef |
| 2018 | Paratyphi B | 7 | 3 | 0 | Single state | Chicken | chicken |
| 2018 | Enteritidis | 4 | 0 | 0 | Single state | Chicken | Chicken, Frozen breaded |
| 2018 | I 4,[5],12:i:- | 18 | 6 | 0 | Multistate | Pork | pork |
| 2018 | Typhimurium | 6 | 0 | 0 | Single state | Beef | jerky |
| 2018 | Typhimurium | 35 | 3 | 0 | Single state | Pork | Pulled Pork |
| 2018 | Adelaide | 29 | 4 | 0 | Multistate | Pork | pork |
| 2018 | Hadar | 17 | 8 | 0 | Multistate | Turkey | turkey |
| 2018 | Typhimurium | 24 | 14 | 0 | Single state | Pork | Pulled Pork |
| 2018 | I 4,[5],12:i:- | 25 | 14 | 0 | Single state | Pork | pork, BBQ |
| 2018 | Enteritidis | 15 | 1 | 0 | Single state | Chicken | chicken, smoked |
| 2018 | Newport | 9 | 1 | 0 | Single state | Turkey | turkey, smoked |
| 2018 | I 4,[5],12:i:- | 3 | 0 | 0 | Single state | Turkey | turkey |
| 2018 | Schwarzengrund | 7 | 1 | 0 | Multistate | Turkey | Ground turkey |
| 2019 | Infantis | 85 | 14 | 0 | Single state | Pork | carnitas, unspecified |
| 2019 | Blockley | 95 | 3 | 0 | Multistate | Chicken | chicken, rotisserie |
| 2019 | Enteritidis | 5 | 2 | 0 | Single state | Chicken | chicken |
| 2019 | Enteritidis | 52 | 10 | 0 | Multistate | Chicken | chicken |
| 2019 | Enteritidis | 23 | 10 | 0 | Multistate | Chicken | chicken |
| 2019 | Enteritidis | 72 | 22 | 0 | Multistate | Chicken | chicken |
| 2019 | Newport | 6 | 3 | 0 | Single state | Beef | steak, prime rib |
| 2019 | Reading | 9 | 2 | 0 | Single state | Turkey | turkey |
| 2019 | Enteritidis | 22 | 8 | 0 | Multistate | Chicken | chicken |
| 2019 | Heidelberg | 28 | 10 | 1 | Multistate | Chicken | chicken |
| 2019 | Infantis | 14 | 5 | 0 | Multistate | Chicken | chicken |
| 2019 | Berta | 30 | 6 | 0 | Multistate | Pork | pork |
| 2019 | Typhimurium | 48 | 13 | 0 | Multistate | Beef | ground beef |
| 2019 | Enteritidis | 9 | 0 | 0 | Single state | Chicken | Chicken Fingers |
| 2019 | Thompson | 15 | 2 | 0 | Single state | Chicken | chicken |
| 2019 | Dublin | 13 | 9 | 1 | Multistate | Beef | ground beef |
| 2019 | Uganda | 18 | 1 | 0 | Single state | Beef | deli meat, sliced roast beef |
| 2019 | Anatum | 8 | 1 | 0 | Single state | Turkey | turkey |
| 2020 | Enteritidis | 6 | NA | 0 | Single state | Chicken | Grilled chicken |
| 2020 | Oranienburg | 18 | 2 | 0 | Single state | Beef | ground beef |
| 2020 | Kiambu | 8 | 1 | 0 | Single state | Beef | kitfo |
| 2020 | I 4,[5],12:i:- | 41 | 4 | 0 | Single state | Pork | whole pig |
| 2020 | Muenster | 4 | 0 | 0 | Single state | Pork | pork |
| 2020 | Enteritidis | 30 | 11 | 0 | Multistate | Chicken | chicken |
| 2020 | Enteritidis | 4 | 2 | 0 | Single state | Chicken | chicken |
| 2020 | Muenchen | 99 | 4 | 0 | Single state | Pork | meat chili |
| 2020 | Enteritidis | 26 | 13 | 0 | Multistate | Chicken | chicken |
| 2020 | Hadar | 34 | 4 | 0 | Multistate | Turkey | Ground turkey |
| 2021 | Enteritidis | 36 | 12 | 0 | Multistate | Chicken | frozen stuffed chicken |
| 2021 | Enteritidis | 8 | 1 | 0 | Single state | Chicken | chicken |
| 2021 | Braenderup | 10 | 2 | 0 | Multistate | Chicken | chicken |
| 2021 | Enteritidis | 50 | 14 | 0 | Multistate | Chicken | chicken |
| 2021 | Enteritidis | 4 | 1 | 0 | Single state | Turkey | deli meat, sliced turkey |
| 2021 | Enteritidis | 87 | 1 | 0 | Single state | Chicken | Chicken, frozen |
| 2021 | Newport | 76 | 27 | 0 | Multistate | Beef | beef, jerky |
| 2021 | I 4,[5],12:i:- | 36 | 7 | 0 | Multistate | Pork | Salame Sticks |
| 2021 | I 4,[5],12:i:- | 25 | 5 | 0 | Single state | Beef | beef, raw |
| 2021 | Newport | 5 | 2 | 0 | Single state | Beef | beef |

*Centers for Disease Control and Prevention (CDC). National Outbreak Reporting System. Atlanta, Georgia: U.S. Department of Health and Human Services, CDC. Download date May 14^th^, 2024. NORS is a dynamic system, and reports can be modified when new information is available, even months or years after data have been closed out. The dataset accurately represents the data present in the system on the date of download and are subject to change.
